# Demonstration of Herpes Simplex Virus, Cytomegalovirus, and Epstein-Barr Virus in Colorectal Cancer

**DOI:** 10.22045/ibj.2016.08

**Published:** 2016-11

**Authors:** Sahar Mehrabani-Khasraghi, Mitra Ameli, Farzad Khalily

**Affiliations:** 1 Department of Microbiology, Tonekabon Branch, Islamic Azad University, Mazandaran, Iran; 2 Department of Medicine, Tonekabon Branch, Islamic Azad University, Mazandaran, Iran; 3 Gastroenterology and Hepatology Research Center, Karaj University of Medical Sciences and Health Services, Alborz, Iran

**Keywords:** Colorectal cancer, Herpes simplex virus, Cytomegalovirus, Epstein-Bar virus, PCR

## Abstract

**Background:**

The present study sought to investigate molecular evidence for association between the presence of herpes simplex virus (HSV), cytomegalovirus (CMV), and Epstein-Barr virus (EBV) in CRC and colorectal polyp by using the PCR method in Iran.

**Methods:**

In this analytical case-control study, we selected 15 patients with CRC, 20 patients with colorectal polyp, and 35 patients without malignancy as controls. After DNA extraction, PCR was used to determine HSV, CMV, and EBV genome by specific primers. Statistical analysis was performed using χ^2^ tests.

**Results:**

Our findings demonstrated that there is no direct molecular evidence to support the association between HSV, CMV, and EBV and human colorectal malignancies.

**Conclusion:**

The results from this study do not exclude a possible oncogenic role of these viruses in neoplastic development of colon cells.

## INTRODUCTION

Colorectal cancer (CRC) is the most common gastrointestinal cancer and the leading cause of cancer deaths in Iran[1]. According to the World Health Organization (WHO), approximately 875,000 new cases of CRC are diagnosed annually worldwide[2]. In general, most CRCs are immunologically silent tumors, grow slowly and often do not produce symptoms until they reach a large size. The incidence of CRC varies throughout the world, with higher frequencies in America, North-Western Europe, Australia, Japan, China, Singapore, and Canada and lower frequencies in African and Asian countries, including Iran[3]. Regardless of the etiology, the majority of CRCs have been demonstrated to arise from adenoma polyps[4]. There are three forms of adenomatous polyps, including tubular histology, villus, and tubular villus. Although a variety of risk factors, such as viral infections, have been found to involve in development of CRC, inherited genetic pre-disposition and molecular mechanisms related to CRC remain under investigation[5,6]. Viral etiologies of human malignancies are an intriguing subject for basic researchers and clinicians. With the exception of hepatitis C virus, all known human tumor viruses contain DNA as their genetic material[7]. Herpes simplex virus (HSV), cytomegalovirus (CMV), and Epstein-Barr virus (EBV) are ubiquitous herpes viruses that infect and establish persistent infections in the host. A potential role of HSV, CMV, and EBV in human carcinogenesis has also been investigated in a variety of studies[8-16]. Available data from clinical studies have so far provided contradictory results, some of which were able to detect the DNA of these viruses in colorectal adenocarcinomas by different laboratory techniques, such as *in situ* hybridization and PCR[13,15,16]. In contrast, others failed to demonstrate the presence of these viruses in tissue samples of CRC, even using the same detection methods[8-10,12,14]. Considering the importance of CRC as the most common gastrointestinal cancer and the possible role of oncogenic viruses in tumorigenesis, the present study aimed to investigate the prevalence of HSV, CMV, and EBV in CRC patients and patients with colorectal polyps by the PCR technique in comparison with healthy subjects.

## MATERIALS AND METHODS

This analytical case-control study was conducted on a total of 35 subjects, including 15 patients with CRC and 20 patients with colorectal polyp. A written informed consent was received from all patients admitted to the Endoscopy Clinic of Toos and Firoozgar Hospitals (Tehran, Iran) between January 2013 and June 2013. Two tissue samples were obtained from each patient, one from malignant tissue and the other one from normal colorectal tissue in an area located 15 cm away from the malignant tissue. In addition, 35 samples from patients without malignancy were used as a negative control. Tissue fragments were sampled by endoscopic biopsy, and an average tissue weight of 25 mg was calculated for each patient. All collected tissues were kept frozen at -20°C until analysis.

DNA was extracted using the KiaSpin® Tissue Kit (Kiagen CA, Iran) according to the manufacturer’s instructions. DNA concentrations were determined from absorbance values at a wavelength of 260 nm using a Biophotometer System (Eppendorf, Germany). The ratios of absorbance at 280/260 nm and 230/260 nm were used to assess the purity of DNA.

PCR amplification of the human *β-globulin* gene was carried out to monitor the quality of the extracted DNA. The identification of HSV, CMV, and EBV genomes was performed according to Zaravinos *et al*.[17] using the specific primers shown in Table 1. PCR amplification was performed in a final volume of 20 µL, containing 10 µL 2X Prime Taq Premix (Kiagen CA, Iran), 3 µL sterile distilled water, 1 µL each forward and reverse primer (TAG Copenhagen, Denmark) and 5 µL DNA template. The human *β-globulin* gene as well as HSV, CMV, and EBV genomes were amplified under the slightly different conditions. After an initial denaturation at 95ºC for 5 min, PCR thermal cycles were set as follows: 35 cycles of denaturation at 95ºC for 50 s, annealing at 55ºC for 45 s, extension at 72ºC for 40 s and a final elongation at 72ºC for 5 min; 35 cycles of denaturation at 95ºC for 50 s, annealing at 64ºC for 45 s, extension at 72ºC for 40 s and a final elongation at 72ºC for 5 min; 35 cycles of denaturation at 95ºC for 50 s, annealing at 60ºC for 45 s, extension at 72ºC for 40 s and a final elongation at 72ºC for 5 min, and 35 cycles of denaturation at 95ºC for 40 s, annealing at 65ºC for 40 s, extension at 72ºC for 40 s and a final elongation at 72ºC for 5 min. Then 5 µL PCR products was analyzed by electrophoresis on a 1.5% agarose gel.

**Table 1:** The nucleotide sequences and length (bp) of the primers used in this study

Primer	Sequence (5´-3´)	Size (bp)	Tm	References
b _2_- F b _2_- R	TCCAACATCAACATCTTGGT TCCCCCAAATTCTAAGCAGA	106	53.2 55.3	17
HSV-1/2 F HSV-1/2 R	CAGTACGGCCCCGAGTTCGTGA TTGTAGTGGGCGTGGTAGATG	465	65.8 59.8	17
CMV-F CMV-R	GTCACCAAGGCCACGACGTT TCTGCCAGGACATCTTTCTC	167	61.4 57.3	17
EBV-F1 EBV-R1	GTGTGCGTCGTGCCGGGGCAGCCAC ACCTGGGAGGGCCATCGCAAGCTCC	102	74.5 71.2	17

Statistical analysis was performed using the SPSS 20 software package (SPSS, Inc., Chicago, USA). The relationship between the prevalence of HSV, CMV, and EBV, and the occurrence of CRC and colorectal polyps was investigated with regards to the location of the samples and compared with the control group. Tissue samples were analyzed using *t* and χ^2^ tests. The results were considered to be statistically significant (*P*<0.05).

## RESULTS

In 15 patients with CRC, HSV DNA was found in tumor samples of 5 individuals (33.3%), and the normal tissue surrounding the tumor also displayed HSV DNA in 5 cases (33.3%). In contrast, no HSV DNA was found in the samples of patients with colorectal polyps (0 out of 20), while 4 (20%) of the patients had HSV DNA only in the normal colorectal tissue surrounding the polyp. HSV DNA was also found in 7 (20%) of 35 patients with non-malignant conditions. Statistical analysis showed that there is no significant association between the prevalence of HSV and the incidence of CRC and colorectal polyps with regard to the location of the samples, as compared with the control group (*P*=0.25).

Of 15 CRC patients, 8 (53.3%) exhibited detectable CMV DNA in their tumor samples, while the normal tissue surrounding the tumor was positive for CMV DNA in 10 cases (66.7%). In 5 patients with CRC (33.3%), CMV DNA was found in both tumor tissue and matched normal tissue. In 20 patients with colorectal polyps, 50% of the samples were positive for CMV DNA, and 70% showed the DNA in the normal surrounding tissue. In 7 patients (35%) with colorectal polyp, CMV DNA was found to be positive for polyp tissue and matched normal tissue. CMV DNA was identified in 13 (37.1%) of 35 patients with non-malignant conditions. Statistical analysis revealed that there is no significant association between the prevalence of CMV and the incidence of CRC and colorectal polyp with regard to the site of the samples in comparison with the control group (*P*=0.28).

In patients with CRC, EBV DNA was found to be positive in 9 (60%) and 4 (26.7%) of 15 samples obtained from the tumor and normal tissue surrounding the tumor, respectively. Two patients (13.3%) with CRC displayed EBV DNA in tumor tissue and matched normal tissue. In 20 patients with colorectal polyps, 7 (35%) had EBV DNA in the polyp samples, while 11 patients (55%) showed EBV DNA in the normal tissue surrounding the polyp. Also, 5 patients (25%) with colorectal polyps were positive for EBV DNA in polyp tissue and matched normal tissue. EBV DNA was found in 14 patients (40%) of the non-malignant group (35%). Statistical analysis revealed that there is no significant association between the prevalence of EBV, and the incidence of CRC and colorectal polyps with regard to the location of the samples in comparison with the control group (*P*=0.44).

## DISCUSSION

In this study, CRC, colorectal polyp, and non-malignant tissues were investigated for the presence of HSV, CMV, and EBV DNA by the PCR method. In cancerous patients, HSV, CMV, and EBV DNA were found in 33.3%, 53.3%, and 60% of the samples, respectively. However, in patients with colorectal polyps, CMV and EBV DNA but not HSV DNA was found in 50% and 35% of the samples, respectively. In the control group, it was established that 20%, 37.1%, and 40% of the samples were positive for HSV, CMV and EBV DNA, respectively. Nevertheless, data showed that there is no association between the presence of the virus and the occurrence of CRC and colorectal polyps, when compared with the tissues from the control group.

Since the discovery of a viral pathology for Gross murine leukemia, the search for oncogenic viruses has been rapidly increased in human malignancies. Based on the current understanding, it is estimated that approximately 15% of the global cancer burden can be linked to oncogenic viruses[18]. Oncogenic viruses may contribute to human carcinogenesis through genetic instability and induce chromosomal aberrations[19].

Various *in vitro* studies have demonstrated that CMV gene products are able to modulate cell cycle progression and apoptosis by regulating the expression of several important host genes. For example, CMV, infection has been shown to transcriptionally activate the expression of the proto-oncogenes, c-foc, c-jun, and c-myc[10]. Kalejta and Shenk[11] have reported that the CMV UL82 gene product, pp71, stimulates cell cycle progression by inducing protein degradation of another important tumor suppressor Rb and its family members p107 and p130. The possible association of CMV with human colorectal adenocarcinomas was first reported by Huang and Roche[13] who detected CMV DNA in 4 out of 7 colonic adenocarcinomas by membrane complementary RNA-DNA hybridization. Interestingly, CMV DNA was also detected in 1 out of 2 cases of familial adenomatous polyposis, but not in normal colonic tissues from the same patients or control cases of Crohn’s disease. However, in other studies, no evidence of a direct association was found between CRC and human CMV (HCMV) infection[10, 12]. Akintola-Ogunremi *et al*.[10] attempted to examine 23, 65, and 51 cases of colorectal hyperplastic polyps, colorectal adenomas and colorectal adenocarcinomas, respectively using immunohistochemical analysis with two different antibodies. No nuclear HCMV antigen positivity was detected in any of the cases studied. In addition, PCR analysis failed to detect viral DNA in 24 selected cases, showing non-specific cytoplasmic immunostaining. In a study by Bender *et al*.[12] on the presence of HCMV in CRC samples, 6 (11%) of the 56 tested tissue samples were found to be positive for HCMV nested PCR amplification. More precisely, 1 (5%) of 20 cases and 5 (21%) of 24 cases were found to be adenoma and moderately differentiated adenocarcinoma, respectively. Surprisingly, no PCR positivity was obtained in samples from well- and poorly differentiated adenocarcinomas.

EBV can generate transcripts to activate the proto-oncogene *c-Myc*, resulting in disruption of various processes such as metabolism, cell cycle regulation, apoptosis, protein synthesis, angiogenesis, and cellular connections. The role of EBV, as a cauitive agent, has been reported in 4%-18% of gastric cancer cases[20]. Although a great number of similar features have been demonstrated in the histology and pathogenesis between gastric and CRC, there are few studies on the relationship between EBV and CRC. In order to investigate the presence of the EBV DNA, Boguszakova *et al*.[14] examined biopsy specimens from 13 patients with adenocarcinoma of the colon and 10 patients with endoscopic polypectomies for colon adenoma. However, they failed to detect viral DNA in the biopsy specimens tested. To detect the presence of EBV DNA in 186 sporadic CRC cases, Karpinski *et al*.[9] showed that after PCR analysis only 19% of the tumor samples were positive for EBV. These results indicated no association between EBV and sporadic CRC. Taken together, our results confirmed the results of Boguszakova *et al*.[14] and Karpinski *et al*.[9] who reported that there is no correlation between EBV infection and CRC progression. However, in a study done by Ruschoff *et al*.[15], PCR was used to detect EBV DNA in 20 cases of colorectal adenocarcinomas, in which they were able to identify EBV DNA in 3 cases. These findings suggested that EBV might be associated with colorectal tumors. Kim *et al*.[16] investigated the presence of EBV in 20 cases of colorectal adenocarcinomas and found 2 EBV-encoded small RNA-positive cases. Grinstein *et al*.[8] found that EBV might play an oncogenic role in epithelial cancers such as CRC and could be involved in hyperplasia and dysplasia.

In summary, the results of this study did not provide direct molecular evidence to support the association between HSV, CMV, and EBV and human colorectal malignancies. However, these findings do not exclude the possible oncogenic role for these viruses in colon cancer pathology.
